# Recent Advances in Deep Learning for SAR Images: Overview of Methods, Challenges, and Future Directions

**DOI:** 10.3390/s26041143

**Published:** 2026-02-10

**Authors:** Eno Peter, Li-Minn Ang, Kah Phooi Seng, Sanjeev Srivastava

**Affiliations:** School of Science, Technology and Engineering, University of the Sunshine Coast, Petrie, QLD 4502, Australia

**Keywords:** remote sensing, synthetic aperture radar, deep learning, machine learning, image processing

## Abstract

The analysis of Synthetic Aperture Radar (SAR) imagery is essential to modern remote sensing, with applications in disaster management, agricultural monitoring, and military surveillance. A significant challenge is that the complex and noisy nature of SAR data severely limits the performance of traditional machine learning (TML) methods, leading to high error rates. In contrast, deep learning (DL) has recently proven highly effective at addressing these limitations. This study provides a comprehensive review of recent DL advances applied to SAR image despeckling, segmentation, classification, and detection. It evaluates widely adopted models, examines the potential of underutilized ones like GANs and GNNs, and compiles available datasets to support researchers. This review concludes by outlining key challenges and proposing future research directions to guide continued progress in SAR image analysis.

## 1. Introduction

Synthetic Aperture Radar (SAR) is an advanced remote sensing technology, typically mounted on mobile platforms like aircraft or satellites, which generates high-resolution geospatial data through its side-looking observation geometry. The system operates by transmitting electromagnetic waves and analyzing the reflected signals, or backscatter, which is used for surface characterization, object detection, and topographic mapping. This backscatter is processed into two primary image formats: Ground Range Detected (GRD), which provides real-valued amplitude data, and Single Look Complex (SLC), a complex-valued product that preserves both amplitude and phase information for advanced analyses. The functionality of SAR systems is defined by several configurable features, including operation across distinct frequency bands, such as X, C, S, L, and P, which offer a trade-off between penetration capability and spatial resolution. For example, the long-wavelength L-band is ideal for penetrating vegetation and soil, while the short-wavelength X-band provides finer surface detail. Furthermore, SAR offers various observation modes, from the wide-area, lower-resolution ScanSAR to the focused, high-resolution Spotlight mode and the continuous, moderate-resolution Stripmap mode. Finally, polarization, ranging from single (HH or VV) and dual (e.g., HH-HV) to quad-polarization (HH, HV, VH, and VV), provides critical information on target structure and scattering mechanisms, with each mode revealing different surface properties. A summary of the typical applications for different SAR bands is provided in Table 1.

Synthetic Aperture Radar (SAR) significantly advances upon traditional radar systems by using a moving antenna to simulate a much larger aperture, thereby achieving high-resolution imagery. A key operational advantage is its ability to function independently of weather and daylight conditions. This all-weather, day-and-night capability, combined with the vast data streams from satellite platforms, enables diverse and critical applications such as disaster management, military tracking, maritime vigilance, and environmental monitoring like detecting illicit mining or oil spills to protect marine ecosystems. The interpretation of this data relies on a sequence of image processing techniques: classification assigns pixels to predefined categories, segmentation partitions the image into meaningful regions, and detection identifies specific objects or targets. However, this analysis is challenged by inherent image coarseness and speckle noise, which can lead to misclassification. The entire analytical pipeline, therefore, involves a critical process of feature extraction, applying sophisticated algorithms, and rigorous training and validation on datasets to ensure assessment accuracy.

The field of SAR image analysis is dominated by two paradigms: Traditional Machine Learning (TML) and Deep Learning (DL). Initial methods relied on TML techniques, such as Support Vector Machines and Random Forests, which required handcrafted features and offered limited learning capacity. Recently, however, DL models like Convolutional Neural Networks (CNNs) and Autoencoders have become predominant. Their ability to automatically learn high-level features from complex raw data has allowed them to overcome long-standing challenges, leading to superior results in object detection, segmentation, and classification. This advancement has unlocked powerful real-world applications, from monitoring geographic changes and detecting forest fires to enhancing maritime surveillance and uncovering illegal mining activities [2,3,4].

While several surveys on deep learning for SAR image analysis exist, the current literature remains fragmented, with most works concentrating on a single facet of the field. For instance, some surveys address specific tasks, such as detection and recognition [2], classification [5], or denoising [6]. Others are confined to particular applications, like ship detection or automatic target recognition [7,8]. Further narrowing the scope, some reviews focus exclusively on a single model type, such as Convolutional Neural Networks [9]. Moreover, comprehensive summaries of available datasets are notably lacking in these works. A summary of these recent surveys and their respective foci is provided in Table 2.

**Figure 1:**
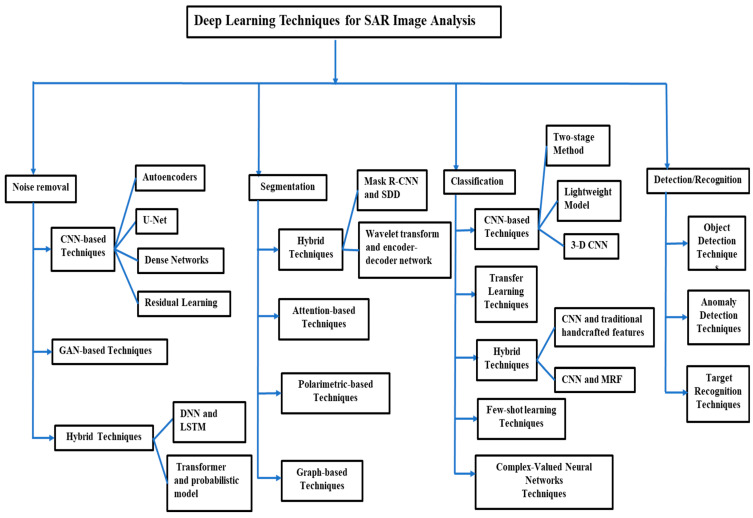
Taxonomy of deep learning techniques for SAR image analysis.

This survey directly addresses these gaps by presenting a unified and comprehensive examination of recent deep learning advancements across the entire spectrum of SAR image analysis. We synthesize progress in fundamental methods, including despeckling, classification, segmentation, and detection, and critically evaluate a diverse range of learning models, from the widely adopted to the underutilized. A key resource we provide is a curated list of available SAR datasets with direct links for researcher access. Finally, we discuss persistent challenges and articulate clear future research directions. The taxonomy of our survey is illustrated in Figure 1.

(1) We provide an extensive review of state-of-the-art deep learning techniques for SAR image analysis, covering a wide range of methods, including despeckling, noise removal, segmentation, classification, and detection.
(2) We discuss various deep learning models, highlighting both widely used and underutilized models in SAR image analysis. This includes an in-depth examination of models such as autoencoders, convolutional neural networks, stacked recurrent neural networks, and deep belief networks, explaining their applications and effectiveness in different SAR tasks.
(3) We present a detailed taxonomy of the survey on deep learning for SAR image analysis, categorizing the various methods covered in the paper.
(4) We compile and highlight available SAR datasets, providing researchers with valuable resources and links. This compilation aims to facilitate access to high-quality data, which is crucial for training and evaluating deep learning models.
(5) We provide insights into key challenges in SAR image analysis, including the difficulty of focusing on relevant features and minimizing redundancy, edge detection errors, high computational demands, the limited availability of labeled training data, the lack of model adaptability, difficulties in handling temporal changes, and real-time implementation constraints. Furthermore, we discuss promising future research directions and potential deep learning approaches to address these challenges.

In summary, this survey synthesizes the current landscape of deep learning in SAR image analysis, encompassing over 128 works to guide readers through the field’s rapid progress. The paper is structured to systematically explore this domain: Section 2 provides a foundational overview of relevant deep learning techniques. Section 3 examines the landscape of deep learning models, from established to novel architectures. Section 4 compiles critical dataset resources for researchers. Section 5 analyzes persistent challenges and proposes future research trajectories, leading to the concluding remarks in Section 6.

## 2. Overview of Deep Learning Techniques for SAR Image

The Deep learning (DL) has established itself as the leading paradigm in SAR image analysis, a direct result of its transformative impact on computer vision. Its key advantage over traditional machine learning is the ability to automatically learn high-level, discriminative features directly from complex SAR data. This capability for end-to-end learning has led to superior performance in critical tasks like segmentation, classification, and detection. The SAR analysis pipeline is inherently sequential and interdependent: successful despeckling is a prerequisite for accurate segmentation, which itself is fundamental to reliable classification and detection. This section reviews the DL methodologies that power this pipeline, whose overall taxonomy is illustrated in Figure 1. Table 3 summarizes SAR image techniques, while Table 4 presents SAR image applications.

A. Deep Learning for Noise Removal in SAR Images

Denoising is a crucial preprocessing stage in SAR image techniques to optimize deep learning efficacy. SAR images often face limitations such as speckle noise and complex feature extraction. Despite providing continuous data acquisition and detailed spatial information, SAR sensors are prone to speckle noise [21]. This noise complicates the analysis and interpretation of large-scale SAR images, affecting deep learning’s predictive accuracy and reducing its applicability in remote sensing. Speckle noise degrades SAR image quality [22], making speckle reduction an essential preprocessing step for most SAR applications. Researchers have developed deep learning techniques for despeckling, which removes noise without blurring edges. These techniques map speckled to clean SAR images, though real-world clean SAR images are rarely available. Techniques such as the Lee filter, enhanced Lee filter, and supervised denoising strive to balance noise reduction and feature retention [23]. An ideal technique smooths noise while preserving features. Deep learning for noise removal can be grouped into the following categories: CNN-based approaches, GAN-based approaches, and hybrid approaches.

(1) CNN-Based Approaches

CNNs have been widely utilized by researchers due to their ability to learn complex patterns and features from data. CNN-based approaches for SAR image despeckling have demonstrated considerable potential, owing to their capacity to effectively learn complex noise patterns and image features. The CNN-based techniques for noise removal are grouped into the following categories based on their architectural design:

Autoencoders: Autoencoders have been extensively utilized for despeckling in SAR images. Autoencoders comprise an encoder and a decoder. Many CNN-based despeckling models employ an encoder–decoder structure, wherein the encoder captures hierarchical features from the noisy image, while the decoder reconstructs the clean image. Qianqian et al. [24] proposed a convolutional denoising autoencoder (C-DAE) for effective reconstruction of speckle-free images. Frontera-Pons et al. [25] utilized denoising autoencoders to mitigate the effects of speckle fluctuations.

U-Net: U-Net is a popular encoder–decoder architecture with skip connections, widely used for despeckling. The skip connections help preserve spatial details by combining low-level and high-level features. Zhang et al. [22] presented the multiscale dilated residual U-Net (MDRU-Net) for SAR image despeckling, which comprises an encoder, decoder, and dilation residual skip connections (DRS). The encoder extracts features, the decoder restores clean images, and DRS reduces differences between shallow and deep features. MDRU-Net may be trained with noisy image pairs and uses five multiscale dilated convolution (MDC) modules to protect image features. Lattari et al. [26] developed a deep encoder–decoder CNN architecture based on U-Net to enhance filtering and texture preservation in SAR images.

Dense Networks: In Dense Networks, every layer is interconnected with all other layers in a feed-forward manner, facilitating feature reuse and improving despeckling performance. The dense networks are characterized by dense connections between layers, with each layer receiving input from all preceding layers. The Dense network is typically organized into dense blocks. Yuan et al. [27] introduced a self-supervised dense dilated convolutional neural network (BDSS) for blind SAR image despeckling, effectively removing speckles while preserving image features without prior knowledge of the image’s properties. BDSS includes a convolutional layer for low-level feature extraction, three dense blocks for high-level feature extraction, and bottleneck and reconstruction layers for output generation. A key limitation of the BDSS method is that the dense and dilated convolutional layers require significant processing power and memory.

Speckle in SAR images is commonly modeled as multiplicative random noise. The speckled image is given by

(1) Y=X ʘ F

where X is the noise-free image, F is the multiplicative random noise, and ʘ indicates element-wise multiplication. Under the assumption of fully developed speckle, F follows a Gamma distribution with the probability density function

(2) $$p(F)=\frac{1}{\Gamma (L)}L^{L}F^{L-1}e^{-LF},\;\;\;\;\;\;F\geq 0$$

where Γ(•) denotes the Gamma function and L represents the equivalent number of looks (ENL).

Residual Learning: Residual learning networks are commonly utilized, where the network learns the variation between noisy and clean images rather than the clean image directly. A novel method, SAR image despeckling using Convolutional Neural Networks (SID-CNN), was proposed by [28]. SID-CNN employs residual learning with convolutional networks to enhance SAR image quality, retaining important features during despeckling and achieving high restoration quality and computational efficiency. Liu et al. [29] introduced a multiscale residual dense dual attention network (MRDDANet) for the denoising of SAR images, ensuring the preservation of the important texture features. The MRDDANet employs multiscale modules with varying kernel sizes to extract shallow features from the noisy SAR images. The shallow features are then passed to a residual dense dual-attention network, which captures deeper features, applying dense connections to fully extract image information. The dual attention mechanism enables the model to focus on both noise suppression and texture preservation. Finally, the denoised image is generated by learning the residual, which helps improve the denoising process.

(2) GAN-Based Approaches

GANs are used for despeckling SAR images owing to their capacity to model complex data distributions and produce high-quality images. Feng Gu et al. [30] introduced a GAN-based approach for despeckling the SAR images. Homogeneous regions are manually selected, after which the GAN learns the speckle distribution to generate realistic-looking images, while a CNN is designed to specialize in speckle removal. Wang et al. [31] introduced an Image Despeckling Generative Adversarial Network (ID-GAN) designed to remove speckles from noisy input images. The ID-GAN is trained using Euclidean, Perceptual, and Adversarial loss. Newey & Sharma [32] presented a GAN for speckle reduction while preserving important image features. This method requires only knowledge of noise statistics for training, not paired images.

(3) Hybrid Approaches

Hybrid approaches for despeckling in SAR images combine multiple techniques to leverage their individual strengths and mitigate their weaknesses. These hybrid approaches help to achieve a balance between removing speckle noise and preserving important image features effectively. To improve SAR image quality, Mohan et al. [33] introduced the Deep Neural Network-based Speckle Noise Removal Technique (DNN-SNRT), which uses convolutional and Long Short-Term Memory (LSTM) neural networks. This method involves preprocessing, deep neural network application, and speckle noise removal. Traditional techniques fail to eliminate speckle noise while preserving features. Zhou [34] presented a speckle reduction method combining Hyperspectral and SAR images using the coherent portions of MNF transformation. The Correlation Simulating Analysis Model (CSAM)-based Spectral Recognition Spatial Smooth Hyperspectral Filter (SRSSHF) smooths the image, eliminates noise, and preserves features. Pan et al. [35] proposed a model that integrates the Swim Transformer and the denoising diffusion probabilistic model (DDPM) for SAR speckle removal. This combination leverages the global feature extraction capability of Transformers and the generative strength of diffusion models to effectively suppress speckle while preserving image details. Liu et al. [36] introduced FRANet, a deep learning-based learning network for SAR image denoising. The model employs a feature refinement network to extract informative features and accelerate training, followed by a feature attention encoder–decoder network with an asymmetric structure to expand the receptive field and enhance feature extraction while reducing parameters. The final denoised image is obtained through global residual learning.

B. Deep Learning for Sar Image Segmentation

Segmentation techniques are crucial for evaluating meaningful parts of SAR imagery, which is challenging due to their high resolution and complexity. Segmentation divides SAR images into small areas, each with unique features. Deep learning approaches yield high accuracy and fast results. Deep learning for SAR image segmentation can be grouped into the following categories.

(1) Hybrid Techniques

Hybrid techniques for segmentation in SAR images improve accuracy, robustness, and computational efficiency by combining multiple approaches to leverage the strengths of each method while mitigating their individual limitations. Yayla & Sen [37] introduced a technique combining a deep mask region convolutional neural network (Mask R-CNN) and sparsity-driven despeckling (SDD) for SAR image segmentation. Mask R-CNN excels at separating meaningful areas and provides precise predictions, while SDD smooths speckle noise and edges. Mask R-CNN is built on the R-CNN, Fast R-CNN, and Faster R-CNN algorithms. Arisoy & Kayabol [38] introduced a mixture-based superpixel segmentation method (MISP) for SAR images, which addresses the limitations of optical image superpixel methods by using SAR image amplitudes and pixel coordinates, resulting in superpixels with regular shapes and smooth boundaries. Wei et al. [39] introduced a hybrid deep learning framework, combining wavelet transform for feature enhancement, an encoder–decoder network for structural feature learning, a cascaded encoder–decoder structure for post-processing refinement, and a self-distillation module to improve hierarchical semantic learning. Experimental results demonstrate that the method effectively enhances segmentation accuracy and boundary clarity.

(2) Attention Mechanism Techniques

Attention mechanisms in SAR image segmentation improve the performance of the models by focusing on the most relevant parts of the image. Yue et al. [40] proposed the attention fully convolutional network (AFCN) for SAR image segmentation, featuring a multiscale attention network (MANet) for improved feature extraction through multiscale, channel, and spatial attention. The approach includes image processing with small slices, segmentation results from AFCN, and a fully connected conditional random field (CRF) to enhance performance. A novel loss function combining cross-entropy losses and Lovasz-Softmax maximizes intersection over union (IoU) and pixel classification accuracy. Experimental results show that the AFCN method effectively improved the segmentation accuracy of the SAR images.

(3) Polarimetric-Based Techniques

Polarimetric-based techniques for segmentation in SAR images are characterized by their use of polarimetric information derived from the scattering properties of targets. These techniques leverage the unique scattering properties of targets to improve segmentation accuracy. Additionally, they are effective in distinguishing between different land cover types or objects. Jing et al. [41] presented a polarimetric space reconstruction network for PolSAR image semantic segmentation, focusing on polarimetric data to improve segmentation accuracy. This method enhances the extraction process by using a spatial amplification coding technique for polarimetric and scattering matrices, a statistics enhancement module for better feature mining, and a dual self-attention mechanism to capture complex relations between matrix elements.

(4) Graph-Based Methods

Graph-based approaches for segmentation in SAR images are characterized by their ability to model the image as a graph, where pixels or regions are represented as nodes, and edges represent relationships or similarities between these nodes. Ma et al. [42] combine an attention mechanism layer with Graph Convolutional Networks (GCNs) to focus on relevant nodes by assigning different coefficients to nodes in a neighborhood for super-wise segmentation in SAR imagery. The GCN processes graph-structured data, while the attention mechanism helps focus on the most relevant nodes, reducing the impact of noisy information.

C. Deep Learning for Sar Image Classification

SAR image classification is crucial in many applications, with deep learning techniques enhancing its effectiveness. Traditional SAR image classification methods are time-consuming due to manual involvement. CNNs have shown remarkable performance in this field. Generally, classification assigns each pixel, or object in an image to a predefined category. It operates on the entire image or predefined regions and does not require explicit object localization. Deep learning approaches for SAR image classification can be grouped into the following categories.

(1) CNN-Based Approaches

CNNs are a popular approach for classification in SAR images due to their ability to automatically learn hierarchical features from data. The CNN-based approaches for classification in SAR images can be grouped as follows:

Two-stage Method: The two-stage method is a significant category under CNN-based techniques for SAR image classification. By dividing the task into feature extraction and classification, these methods achieve higher accuracy in SAR image classification. Li et al. [43] introduced a two-stage method using CNN for classification and metric learning, enhancing classification accuracy. The first stage involves standard SAR image classification and feature extraction, while the second stage trains a relational network for metric learning. This method, tested on the OpenSARShip and MSTAR datasets, achieved higher classification accuracy than standard CNNs in military target classification.

Lightweight Model: The lightweight model has gained significant attention in CNN-based approaches for SAR image classification due to its ability to achieve high performance with reduced computational complexity and memory usage. Passah & Kandar [44] developed a lightweight model with four Convolution Depthwise Convolution Activation Maxpooling (CDCAM) blocks, one Convolution Activation Maxpooling (CAM) block, a fully connected layer, two dense layers, a dropout layer, and two activation layers, improving computational efficiency.

3-D CNN: 3-D CNNs are an extension of traditional 2-D CNNs, designed to handle SAR images with multiple channels or polarizations. Zhu et al. [45] implemented a 3-D CNN for target classification using raw SAR images, luminance contour, and segmentation to create a 3-D input, achieving high classification accuracy on the MSTAR dataset. The 3-D CNN improves on the performance of traditional 2-D CNNs by capturing spatial and channel-wise features simultaneously.

(2) Transfer Learning Approaches

Transfer learning is widely used in CNN-based classification for SAR images because SAR data often suffers from limited labeled datasets and challenges such as speckle noise and complex scattering mechanisms. Transfer learning involves using CNN models pre-trained on large-scale datasets from a different domain. He et al. [46] used a bilinear CNN to classify ships from SAR images, leveraging polarization information with a dual-stream CNN framework. Bilinear pooling on deep CNN features from two single-polarization SAR images enhances ship representation discrimination. Fine-tuning pre-trained CNN models with a large-scale SAR ship detection dataset addresses the limitations of small-scale datasets.

(3) Hybrid Approaches

Hybrid approaches for the classification of SAR images combine multiple techniques to leverage their complementary strengths, addressing the challenges posed by SAR data, such as speckle noise, complex scattering mechanisms, and high dimensionality. The hybrid approach integrates methods from different domains, such as machine learning, deep learning, and traditional image processing, to improve classification accuracy. Nehary et al. [47] combined a CNN with traditional handcrafted features (e.g., Kaze features, HOG, LBF, BF, and SIFT) for better ship classification, achieving higher sensitivity, precision, F1-scores, and accuracy. Yang et al. [48] proposed an algorithm combining a CNN and the Markov random field (MRF) to enhance spatial relationships between superpixels, mitigating pixel-level and region-level misclassification. The method starts with a CNN to initialize region labels and then creates a probability field to enhance spatial relationships between superpixels. A region-level MRF model classifies the superpixels, integrating intensity and probability fields. This approach mitigates pixel-level misclassification and corrects region-level misclassification by improving spatial descriptions. The CNN extracts pixel-level features, while the simple linear iterative clustering (SLIC) algorithm generates superpixels. Fang et al. [49] introduced a hybrid technique that leverages the strengths of self-attention, selective kernel attention, and an encoder–decoder network to improve the classification accuracy in PolSAR images.

(4) Few-Shot Learning

Few-shot learning in SAR image classification is characterized by the ability to learn and generalize from a very limited number of labeled examples. This approach is particularly useful for SAR image classification, where acquiring large amounts of labeled data is challenging, expensive, or time-consuming. Cai et al. [50] proposed an improved Prototypical Network (PN), called ST-PN, which incorporates a spatial transformer module to perform feature-wise alignment rather than pixel-wise alignment. The ST-PN captures more semantic information, reduces discrepancies caused by different observation angles, and lowers the computational cost by using fewer parameters in deeper layers.

(5) Complex-Valued Neural Networks (CVNNs)

SAR images are inherently complex-valued because they contain both magnitude and phase information, which makes CVNNs a natural fit for processing SAR data. CVNNs for classification in SAR images are characterized by their ability to process complex-valued data directly. Huang et al. [51] proposed a deep learning approach (Deep SAR-Net) for complex-valued SAR images, learning spatial texture characteristics and backscattering patterns. Zhang et al. [52] introduced a CVNN for SAR image interpretation that utilizes amplitude and phase information from complex SAR data.

D. Deep Learning for Sar Image Detection and Recognition

Deep learning techniques are increasingly being applied to the analysis of SAR images to enhance detection and recognition performance. Generally, detection focuses on determining the presence and location of potential targets in SAR images. It separates targets from background clutter, such as speckle noise or terrain backscatter, whereas recognition identifies the detected targets. Recognition assigns a semantic label, such as a ship type, to each detected object based on its scattering characteristics and spatial features. Researchers have utilized deep learning for various SAR image detection applications, including ship and oil spill detection. Deep learning for SAR image detection and recognition is categorized into the following groups.

(1) Object Detection Techniques

Object detection in SAR images is characterized by the need to address unique challenges such as speckle noise, complex backgrounds, and limited labeled data. Ship detection is a key application and a research hotspot within the broader field of object detection. It involves identifying and localizing ships in SAR imagery. Jianwei [53] introduced an improved Faster R-CNN for ship detection, which utilizes ZF-Net, feature fusion, transfer learning, and challenging negative mining, improving the average precision from 70.1 to 78.8. The standard Faster R-CNN framework, while effective for general object detection, exhibits several limitations for SAR ship detection, including difficulty detecting small, low-resolution objects due to region of interest (ROI) pooling from only the final feature map, limited transferability of features learned from optical datasets, and the need for domain-specific parameter adjustments such as anchors, proposals, and dropout rates. To address these issues, the improved Faster R-CNN modifies the original architecture. In the original Faster R-CNN design, the five convolutional layers are followed by a rectified linear unit (ReLU) activation, local response normalization (LRN), and a max-pooling layer. In contrast, the improved architecture applies only a single ReLU activation after the last three convolutional layers, while removing both LRN and conventional max-pooling operations. Spatial downsampling is instead performed by the ROI pooling layer, which effectively replaces max-pooling by aggregating features within each proposed region. Zhang et al. [54] implemented a novel salient feature fusion method, a one-stage ship detection network, which improves SAR image ship detection precision. This method fuses salient and deep CNN features, employing a saliency map extraction algorithm with frequency domain and multi-scale pyramid features. The two-stream network includes an upper-stream network for multi-scale deep CNN features and a lower-stream network for multi-scale salient features. An improved bi-directional feature pyramid network further reduces computational complexity. The technique accurately detects SAR ship images with high precision, offering an end-to-end processing method that differs from traditional multi-step methods.

Zhang et al. [55] introduced HyperLi-Net for high-accuracy, high-speed SAR ship detection with low computational cost. It includes five external modules (CSA, MRF, FF, DC, FP) and four backbones (Backbone-1, -2, -3, -4) for feature extraction. Chen et al. [56] proposed a speckle-free SAR ship detection technique, achieving high performance on Sentinel-1, Gaofen-3, and RADarsat-2 datasets. Zhao et al. [57] developed a ship detection model without pre-training, which uses DetNet and stacked convolutions for small object detection, achieving a 94.7% precision rate. It also incorporates a feature reuse strategy for better parameter efficiency and adds multiple branches for multiscale object detection. Sun et al. [58] introduced BiFA-Y, a YOLO-based SAR ship detector featuring bi-directional feature fusion and angular classification, achieving a precision of 94.85%, a recall of 93.97%, an AP of 93.90%, an F1 of 0.9441, with speed detection of approximately 13.3 ms per 512 × 512 image. Man et al. [59] proposed ELSD-Net, a lightweight and effective SAR ship detector based on the YOLOv5n architecture. The network integrates three key modules: the Nonstride Feature Extraction (NSFE) module to reduce network parameters, the Multiscale Efficient Channel Attention (MECA) module to enhance multiscale feature perception, and the Strip Partial Convolution (SPC) module to improve global modeling with minimal computational cost. Experiments on the SAR Ship Detection Dataset (SSDD) show that ELSD-Net achieves a detection accuracy of 98.2% while requiring only 0.3 M parameters and 1.2 G FLOPs, demonstrating superior efficiency and performance compared to existing methods. Selvam et al. [60] introduced YOLO-SAIL, which replaces the Bi-directional Feature Pyramid Network (Bi-FPN) with a Path Aggregation Network (PAN) for enhanced multi-scale feature extraction, incorporates a Normalized Attention Mechanism (NAM) to focus on dense ship targets, and adds a feature fusion layer to improve detection of smaller ships. Evaluation on the SSDD and High-Resolution SAR image datasets (HRSID) demonstrates that YOLO-SAIL achieves F1-scores of 98.22% and 94.68%, respectively, outperforming most existing methods.

(2) Anomaly Detection

Anomaly detection in SAR image analysis is a technique used to identify unusual or unexpected patterns in data that substantially diverge from the standard. Oil spill detection is a key application and a research hotspot within the wider domain of anomaly detection. Huang et al. [61] introduced Faster R-CNN for the detection of marine oil spills via satellite SAR images, integrating a Regional Proposal Network (RPN) with Fast R-CNN for rapid end-to-end detection. The training, validation, and testing were conducted using 1786 vertical polarisation SAR images from C-band Sentinel-1 and RADARSAT-2. Results show that Faster R-CNN outperforms other methods in both accuracy and robustness under diverse environmental situations. Gong et al. [62] combined CNN, sparse autoencoder (SAE), and unsupervised clustering for ternary change detection, achieving accurate results by transforming the log-ratio difference image and generating pseudo labels. SAE transforms the log-ratio difference image to extract significant changes while reducing noise. CNN then uses these feature maps, and unsupervised clustering generates pseudo labels for training the CNN as a change feature classifier. Doan et al. [63] introduced a deep learning model for floodwater detection in SAR images based on change detection. The model operates on bi-temporal satellite images using a parallel Siamese architecture with a Swin Transformer backbone for multi-level feature extraction. Feature differences are computed to enhance representations before decoding, and the decoder predicts and fuses changes across levels to generate the final flood map. Experimental results demonstrate that the method outperforms existing approaches, achieving a recall of 94.6%, precision of 96.9%, and an F1-score of 95.7%, with a computational cost of 32.3 GFLOPs. Huang et al. [64] introduced a two-stage deep learning approach for the automatic localization and segmentation of oil spills in Sentinel-1 SAR imagery. The approach employs Faster R-CNN for initial oil spill localization, followed by UNet++ for pixel-wise segmentation of the identified regions. Experimental results show that the method achieves high precision and recall of 87.46% and 87.59% for localization, and 89.55% and 89.37% for segmentation. It operates effectively under wind speeds of 2–10 m/s and processes full-resolution SAR images in under one minute, demonstrating robustness and efficiency for operational oil spill monitoring using spaceborne SAR data.

(3) Target Recognition

Target recognition involves identifying objects in an image, typically after detection, and assigning each a specific label. Jia et al. [65] introduced a deep learning fusion recognition approach for SAR imagery target recognition and utilized principal component analysis (PCA) to extract 1-D features as input for a stacked autoencoder (SAE) to extract deep features. They also proposed a method integrating decision-level and feature-level data for different SAR image features. The experimental results showed that this method is adaptive and robust against variations in attitude angle, background, and noise. Yue et al. [66] proposed a semi-supervised CNN technique to improve SAR ATR accuracy with unlabeled samples, using class probabilities and linear discriminant analysis (LDA). The method uses a CNN to obtain class probabilities of unlabeled samples, optimizes these probabilities through thresholding, and uses them for scatter matrix calculation in linear discriminant analysis (LDA). This scatter matrix modifies the CNN’s loss function, enhancing SAR ATR accuracy when labeled samples are insufficient. Zhang et al. [67] introduced a Multiscale Global Scattering Feature Association Network (MGSFA-Net). The network first segments the ship from the background, extract scattering centers (SCs), and converts them into local graph structures using a k-nearest neighbors approach. These structures are enhanced and associated via dedicated modules to produce multiscale global scattering features, which are fused with deep features extracted by a multikernel module. Experimental results on FUSAR-Ship and OpenSARShip datasets demonstrate that MGSFA-Net significantly improves recognition performance, even in few-shot conditions, with accuracy gains of 2–3%. Shi et al. [68] introduced a CNN with a hybrid attention mechanism for target recognition. The model consists of a trunk branch that uses a residual shrinkage network and improved channel attention to extract main features, and a soft branch that utilizes up- and down-sampling to compute mixed attention weights that enhance the input-output mapping. Experimental results on the MSTAR dataset show that the model achieves a recognition rate of 99.6% and demonstrates strong robustness against impulse noise. Shi et al. [69] proposed an attention-based neural network that extracts information in two stages: first by reducing noise to obtain high-level features, and then by applying hybrid attention. The model employs a dual-channel one-dimensional convolution to reconstruct a residual shrinkage network, forming a lightweight and efficient feature module that serves as the backbone of the network. Mixed adaptive pooling and dimensionality adjustment via pooling and linear interpolation are used to generate multi-dimensional feature weights. Experimental results on the MSTAR dataset show that the model significantly reduces parameters and computational complexity while maintaining accuracy and demonstrating strong robustness to noisy images. Zhou et al. [70] introduced a transformer-based encoder–decoder network for flood region recognition in SAR images. The model employs a mix transformer encoder to enhance global feature discrimination, a noise filtering module (NFM) to reduce redundant low-level information and mitigate edge discontinuities, and a multiscale depth-wise convolution module (MDCM) for enhanced multiscale feature representation. Experimental evaluation on the Sen1Floods11 dataset demonstrates that the approach outperforms existing methods in segmentation accuracy and reliability.

## 3. Popular Deep Learning Models in SAR Image Analysis

This section describes popular deep learning models in SAR image analysis, explaining their mechanisms and highlighting recent techniques that have not yet been extensively utilized in SAR. Deep learning models transform input data into meaningful feature representations for specific tasks [14]. These models include generative adversarial networks (GANs), convolutional neural networks (CNNs), deep belief networks (DBNs), autoencoders (AEs), recurrent neural networks (RNNs), and graph neural networks (GNNs).

A. Convolutional Neural Networks (CNN)

Convolutional Neural Networks (CNNs) are extensively used in computer vision tasks owing to their success in the ImageNet Large Scale Visual Recognition Competition (ILSVRC) [71]. CNNs learn low- and high-level features from raw images through convolutional and pooling layers, utilizing these features to tasks like image detection, classification, object recognition, and segmentation [14]. They use local connections to extract 2-D spatial features. CNNs consist of input, convolutional, pooling, and fully connected layers, with configurations varying by application.

Convolutional layers are essential in CNNs, convolving image patches with kernels to produce feature maps. Let X be the input of dimensions (m×n×z), and Y the output, where m×n are the spatial dimensions, z is the number of channels, and $x_{i}$ is the ith feature map of X. With k filters, the jth filter has weight $w_{j}a$nd bias $b_{j}$. The jth output of the convolutional layer is:

(3) $$y_{j}=\sum_{i=1}^{z}f\left(x_{i}*w_{j}+b_{j}\right),j=1,2,\ldots ,k$$

where f(∙) is the activation function that enhances the network’s nonlinearity, and * stands for the convolutional operator.

Pooling layers reduce the spatial dimensions of feature maps produced by convolutional layers, helping to extract more generalized features and minimize redundancy. In turn, this decreases the number of parameters and computational demands, enabling more abstract feature representations. Representing Ws as a neighbouring region of size p × p, the average pooling operation over this region is:

(4) $$P=\frac{1}{F}\sum_{(i,j)\epsilon W_{s}}x_{i,j}\;$$

where F is the number of elements in Ws and $x_{i,j}$ is the activation value at position (i,j).

Fully connected layers process the flattened output from preceding pooling layers. The operation of a fully connected layer is defined as:

(5) $$Y^{\prime }=\sum_{i=1}^{C}f\left(WX^{\prime }+b\right)$$

where $W^{\prime }{,Y}^{\prime },X^{\prime }andb$ represent the weight, output, input, and bias of a fully connected layer.

CNNs have some unique mechanisms and limitations based on their architecture and training. These mechanisms include: local feature extraction, hierarchical learning, and parameter sharing, making them ideal for processing. CNNs process images using small kernels like 3 × 3, 5 × 5 that slide across the image, to focus on local regions. These features allow the network to detect edges, corners, textures and other localized features. This mechanism also allows the CNNs to learn spatial hierarchies, where the early layers capture low-level features, and deeper layers combine them into higher-level representations like objects or scenes. Furthermore, CNNs don’t rely on hand-crafted features; they learn features directly from data. These unique mechanisms make it well-suited for SAR tasks such as denoising, classification, segmentation, and detection. The limitations of CNNs include an inability to capture long-range dependencies, a reliance on large amounts of high-quality training data, and high computational costs. Deep CNNs are particularly resource-intensive, often requiring GPUs and significant memory for both training and inference.

**Table 3:** Summary of SAR Image Technique.

Deep Learning Technique	Application	Dataset	Feature/Focus of Work	Performance Metrics	References
Faster R-CNN	Satellite SAR oil spill discharge detection	1786C-band Sentinel-1 and RADARSAT-2	Focus on achieving rapid end-to-end oil spill detection while maintaining acceptable accuracy.	Intersection-over-Union, Precision, and Recall	[61]
Improved faster R-CNN	Ship detection	SSDD	Focuses on achieving high accuracy and lower test costs for SAR ship detection.	Average Precision and Average processing time per image	[53]
SAR image despeckling with Convolutional Neural Networks (SID-CNN)	Enhancement of SAR image quality	Set 12 and Berkeley segmentation dataset (BSD68). They are Optical datasets used solely to generate synthetically speckled images for training and validating SAR despeckling methods alongside real SAR images	Focuses on removing speckle from SAR images while ensuring high restoration quality and computational efficiency.	Peak Signal to Noise Ratio, and Structural Similarity Index	[28]
HyperLi-Net	Ship detection	SSDD, Gaofen-SSDD and Sentinel-SSDD	Focuses on achieving high accuracy and speed ship detection.	Detection Accuracy, Detection Speed, Number of Parameters, Computational Cost, Model Size	[55]
A lightweight deep learning model	Earth observations	MSTAR	Focuses on computational efficient	Accuracy, Precision, Recall, and F1-Score.	[44]
Deep Mask R-CNN	Image Segmentation	Mstar SAR	Focuses on achieving high accuracy prediction in SAR image segmentation	Accuracy and Loss Rates	[37]
Convolution and Long Short-Term Memory-based neural networks	Enhancement of SAR Image quality	TerraSAR-X	Focuses on removal of speckle noise	Peak Signal to Noise Ratio (PSNR), Standard Deviation (SD), Equivalent Number of Looks (ENL), and Edge Preservation Index (EPI).	[33]
CNN and Traditional Handcrafted features	Ship Classification	OpenSARShip	Focuses on the high accuracy of ship classification.	Sensitivity, Precision, F1-Score, Accuracy	[47]
Improved region convolutional neural network (R-CNN)	Ship detection	Custom SAR ship dataset collected from TerraSAR-X, RADARSAT-2, and COSMO-SkyMed sensors	Focuses on effective and precise detection of ship targets effectively.	Recall, Factor of the Metric (FoM) and mean Average Precision (mAP)	[72]
one-shot learning with Siamese Network	Ship classification	OpenSARShip	Focuses on improving classification accuracy for SAR Ship.	Area under the receiver operating characteristic Curve (AUC), Precision, Threshold, Recall, F1-Score, Parameters, and Model size.	[73]
Adaptive Fuzzy Superpixels (AFS) algorithm for PolSAR images classification.	PolSAR classification	Flevoland, Oberpfaffenhofen, San Francisco Bay	Focuses on reducing misclassification rates	Undersegmentation Error (UE), Pure Superpixel Ratio (PSR), Boundary Recall (BR), Average Accuracy and Kappa	[74]
Adaptive Graph convolutional network for image classification	PolSAR image classification	Oberpfaffenhofen	Focuses on enhancing feature extraction and improving classification accuracy.	Per-Category Accuracy, Overall Accuracy (OA), Average Accuracy (AA), and Kappa Coefficient (KC)	[75]
Multiscale superpixel-guided weighted graph convolutional network	PolSAR image classification	Flevoland, ESAR Oberpfaffenhofen, San Francisco	Focuses on reducing misclassification rates and preserving classification details.	Per-Class Accuracy, Overall Accuracy (OA), Average Accuracy (AA), and Kappa Coefficient.	[76]
Transformer-based network	SAR image despeckling	Set12 dataset	Focuses on reducing speckles while preserving fine details.	Peak Signal-to-Noise Ratio (PSNR), Structural Similarity Index (SSIM)	[77]
Low-frequency and contour sub-bands driven polarimetric Squeeze-and-Excitation network (LC-PSENet)	PolSAR image classification	Flevoland, ESAR Oberpfaffenhofen	Focuses on enhancing feature learning and achieving high classification accuracy.	Overall Accuracy	[78]

B. Recurrent Neural Network (RNN)

Recurrent Neural Networks (RNNs) are deep neural networks with a feedback mechanism [79]. They employ recurrent units that use both the current input and output from the previous state as input [14]. RNNs excel in handling data of varying lengths, such as text and time series [9]. In visual processing tasks like image captioning and future frame prediction, the recurrent unit’s weights can be substituted with convolutional kernels. RNNs are effective in analyzing SAR images by extracting spatial dependencies from image patches.

**Table 4:** Summary of SAR image application.

Application	References
Oil spill discharge detection	[80,81,82,83,84,85,86,87,88,89]
Ship detection	[53,54,56,57,90,91,92,93,94,95]
Automatic target recognition in military operations	[69,96,97,98,99]
Agriculture monitoring	[100,101,102,103,104,105]
Forestry management	[106,107,108,109,110,111]

C. Deep Belief Network (DBN)

Deep Belief Networks (DBNs) are constructed by stacking multiple unsupervised networks, specifically restricted Boltzmann machines (RBMs), with each RBM layer connected to the preceding and subsequent layers [9]. A single RBM, consisting of a visible layer and a hidden layer, has limited feature extraction capability. Combining multiple RBMs enhances this ability, making DBNs effective for learning high-level features from high-resolution SAR images [112].

D. Autoencoder (AE)

Autoencoders reduce dimensionality and extract features using an encoder to extract representative features and a decoder to reconstruct the input [11]. Stacking multiple autoencoders allows each layer to learn increasingly abstract representations, facilitating feature extraction. Stacked autoencoders perform layer-wise unsupervised pre-training, which is later fine-tuned using backpropagation. Types of autoencoders include sparse, convolutional, denoising, and variational autoencoders.

The encoding and decoding functions are shown below:

(6) Y=σ(wX+b)

(7) $$\hat{X}=\sigma (w^{T}X+b^{T})$$

where w represents the weight, X denotes the input, b signifies the bias and Y indicates the encoded output with a non-linear activation function, sigmoid (σ). $\hat{X}$ represents the reconstruction of the input.

E. Generative Adversarial Network (GAN) Based SAR Models

Generative Adversarial Networks (GANs) are powerful and exciting in deep learning due to their ability to model complex data distributions and perform image-to-image transformation. The GANs consist of two components: a generator and a discriminator. The generator creates synthetic data, while the discriminator distinguishes real data from synthetic data [9,113]. Synthetic data can be used to simulate speckle noise along with clean ground truth, making it ideal for training and benchmarking despeckling methods. It can also provide abundant labeled samples, which improves segmentation model training and generalization, especially when real labeled data is scarce, though this depends on how realistic the synthetic data is. Additionally, synthetic data can supplement training sets for classification and help balance class distributions; however, careful domain alignment is necessary to prevent models from learning biases specific to the synthetic data. Generative approaches learn data distribution patterns to generate new data, while discriminative approaches model label dependence on training data [114]. GANs generate new data instances resembling actual data, with both networks trained simultaneously until equilibrium is reached [113]. Since SAR images often suffer from limited data and complex backscatter properties, GANs are valuable for the following:

Realistic SAR data generation: GANs learn to replicate the statistical distribution of real SAR data, including the texture, speckle noise, and geometric patterns.

Data Augmentation: SAR images can be used to train other models when labelled data is scarce.

Cross-modal translation: GANs can convert optical imagery to SAR-like data or vice versa by learning cross-domain mappings.

F. Graph Neural Network (GNN)

Graph Neural Networks (GNNs) analyze and interpret graph-structured data by enabling information exchange between nodes and recognizing dependencies between nodes and edges [115]. GNN models include Graph Convolutional Networks (GCNs), GraphSAGE, and Graph Attention Networks (GATs). Graphs consist of nodes interconnected by edges, which represent relationships and dependencies within complex systems. GNNs preserve the input graph’s connectivity by using the same adjacency list and feature vector count [115]. Mathematically, the graph G=(V,E), where V represents nodes and E represents edges. GNNs are well-suited for SAR because they can handle non-Euclidean data structures such as irregular spatial layouts and connectivity graphs; they also model relational dependencies between entities like ships, buildings, and regions. GNNs enhance SAR image analysis tasks like detection, classification, and segmentation by treating pixels as nodes in a graph. Table 5 presents the suitability of deep learning models, using symbols ✓ indicating which are suitable and ⊠ which are not. GNNs find their application in ship detection and monitoring, scene classification, change detection and structural damage assessment.

G. Transformer

Transformers are deep learning architectures and are widely used in the field of natural language processing (NLP). However, NLP has been revolutionized and is now widely used in tasks like text generation, translation, and even in computer vision and speech processing. A Transformer-based attention mechanism was first introduced to NLP task by [116] for machine translation applications. It uses self-attention layers instead of the traditional recurrent neural network, which struggles to encode the long-range dependencies. The transformer consists of two main components, the encoder and decoder. The transformers have the following advantages: parallelization, long-range dependencies, scalability and versatility (i.e., not only used in NLP). Popular Transformers backbones, such as Vision Transformers (ViTs) and Swin Transformers, have been successfully applied to the SAR image analysis task, yielding significant performance improvements [18,117].

## 4. Datasets for SAR Image Analysis

This section introduces several datasets widely used in machine learning research for detecting and classifying SAR imagery, including the AirSAR’s Flevoland, MSTAR, OpenSARShip, SARFish, SARShip Detection, San Francisco Bay, TerraSAR-X, and FurSAR-Ship datasets.

A. AIRSAR Flevoland Dataset

The AIRSAR Flevoland dataset from the Netherlands is widely used for SAR image classification and segmentation [11]. It is an L-band dataset (1–2 GHz frequency, 30–15 cm wavelength) consisting of 31 SAR images from the Gaofen-3 satellite, featuring various imaging modes (spotlight, strip map) and resolutions (1 m, 3 m). The dataset includes over ten object categories (fishing boats, ships, tankers, etc.) and scene types (islands, ports, reefs, sea surfaces) [14]. It is applied in SAR image techniques for geophysical monitoring, biomass, and vegetation. The AIRSAR system acquires two datasets. Dataset 1, acquired in 1989, has an image size of 750 × 1024 pixels and includes 15 categorized terrain types (potatoes, stem beans, wheat, peas, beet, rapeseed, forest, barley, lucerne, wheat 2, bare soil, grass, wheat 3, water, buildings) [52], totaling 157,296 labeled pixels [118]. Dataset 2, acquired in 1991 from another region of Flevoland, Netherlands, has an image size of 1020 × 1024 pixels and includes 14 labeled terrain categories (potatoes, beet, fruit, onions, oats, rapeseed, barley, peas, wheat, beans, maize, flax, grass, lucerne) [118], totaling 135,350 labeled pixels.

B. Moving and Stationary Target Acquisition and Recognition (MSTAR) Dataset

The MSTAR dataset is an X-band dataset used to evaluate automatic target recognition (ATR) in SAR images. Acquired by the Sandia National Laboratories SAR sensor platform, it serves as a benchmark for ATR models in SAR applications. The MSTAR dataset, widely used for SAR ATR in military land vehicle applications, provides ten classes of targets [96] and contains 17,658 SAR image chips, including an additional class of simple geometric-shaped targets [45].

C. OpenSARShip Dataset

The OpenSARShip dataset is a C-band SAR dataset designed for analyzing Sentinel-1 ship imagery and consists of two classes: OpenSARShip 1.0 and OpenSARShip 2.0. OpenSARShip 1.0 serves as a benchmark for evaluating ship geometric and scattering characteristics, as well as detection and classification methods. It includes 11,346 SAR ship chips from 41 Sentinel-1 images, captured under various environmental conditions, along with automatic identification system (AIS) signals [119]. OpenSARShip 1.0 is characterized by its diversity, extensive scale, reliability, and public accessibility. OpenSARShip 2.0, the latest version, meets more advanced requirements for ship target interpretation, covering 34,528 Sentinel-1 SAR image chips that include ship geometric data, ship type, and associated AIS data [14,119]. It includes interference labeling, a large volume, and type levels. The OpenSARShip datasets, derived from 87 Sentinel-1 images captured under diverse conditions, are in interferometric wide swath (IW) mode and consist of two products: ground range detected (GRD) and single look complex (SLC).

D. SARFish Dataset

The SARFish dataset, comprising C-band SAR imagery, is utilized for the training, validation, and testing of deep learning algorithms aimed at ship classification and detection in complex-valued SAR images. SARFish advances automated ship detection, contextual representation learning, and deep complex neural network applications by utilizing Sentinel-1 data and integrating SLC and GRD imagery from the ESA Copernicus Open Access Hub [120]. It is the only dataset suitable for training deep learning algorithms in full-size dual-polarization SLC and GRD SAR imagery.

E. FUSAR-Ship Dataset

The FUSAR-Ship dataset is a C-band SAR dataset, developed from temporally and spatially aligned Gaofen-3 SAR images and ship AIS signals, contains more than 5000 ship chips with corresponding AIS data [14]. This enables tracking each unique ship. The dataset is characterized by high resolution, consistency, diversity, scalability, and extensibility, and is employed for ship recognition, wake tracking, and semantic segmentation [8].

F. SARShip Detection Dataset

The SAR Ship Detection Dataset (SSDD) is a C-band SAR dataset and is one of the first widely recognized open dataset for advancing deep learning in ship detection from SAR imagery. Comprising 108 Sentinel-1 and 102 Chinese Gaofen-3 images, it has 43,819 ship chips, each measuring 256 × 256 pixels, and serves as a benchmark for small object detection and multi-scale object detectors [14,121]. Research shows SSDD is preferred in 46.59% of 161 public reports for studying DL-based SAR ship detection models [122], highlighting its influence in SAR remote sensing.

G. San Francisco Bay Dataset

The San Francisco Bay dataset covers four targets: low-density urban, vegetation, high-density urban, and water. One L-band version contains 900 × 1024 pixels with a 10 m spatial resolution [123,124], widely used in PolSAR image classification research. Another C-band version, captured by a RADARSAT-2 sensor, has a resolution of 10 × 5 m and an image size of 1800 × 1380 pixels [125]. This dataset is useful for developing multi-scale and small object detectors.

H. TerraSAR-X Dataset

The TerraSAR-X dataset, obtained from Single-look Slant-range Complex (SSC) data of the TerraSAR-X satellite in imaging mode, focuses on man-made targets. Its high resolution reveals the detailed structures of these targets [51]. Accessing these SAR datasets for deep learning simulations is challenging. Table 6 provides available datasets and links for future researchers to access them easily.

## 5. Research Challenges and Future Directions

Despite the significant advances enabled by deep learning, SAR image analysis remains a challenging field with considerable room for improvement. Critical limitations persist, including poor feature selectivity leading to computational redundancy, difficulties in precise edge detection, and a heavy reliance on large, labeled datasets. Furthermore, models often lack adaptability across diverse scenarios, struggle with temporal changes, and face constraints in real-time deployment. This section analyzes these key challenges and proposes targeted directions for future research to overcome them.

A. Feature Focus and Redundancy

Many SARs analysis techniques struggle to focus on relevant features, leading to redundancy and less accurate results. Inspired by human vision, the attention mechanism enables CNN models to focus on relevant details for the task [130]. This mechanism enhances feature representation in image extraction by directing the network’s focus [131]. In SAR image analysis, utilizing the attention mechanism will significantly enhance the performance of the model by allowing the model to focus on the most relevant features in the image while ignoring less important parts. Various attention mechanisms, such as channel attention, self-attention, and spatial attention, are widely used in remote sensing to focus on relevant image features [109,125,132]. Each attention mechanism serves a different purpose in SAR image analysis. The self-attention mechanism considers relationships between image parts and calculates attention weights, capturing long-range dependencies and global context. For instance, self-attention-based transformers can be integrated into deep learning networks to capture local and global relationships, improving SAR image classification. Self-attention is used in transformer-based networks.

Spatial attention enables the model to focus on important spatial regions within an image for classification, assigning higher weights to relevant areas and lower weights to irrelevant ones. Since SAR images have different channels with varying importance, channel attention helps the model focus on the most informative channels, enhancing classification performance. An example of a model using channel attention is Squeeze and Excitation (SE). Utilizing attention mechanisms in SAR image analysis focuses on relevant image parts and eliminates redundancy, improving SAR image analysis accuracy. These mechanisms reduce redundant information by learning spatial transformations. Future SAR image analysis should integrate attention mechanism models into deep learning networks to enhance performance by focusing on the most relevant features. Additionally, combining spatial and channel attention in deep learning networks can improve analysis accuracy by targeting the most relevant spatial regions and channels.

B. Availability of labeled training data

Deep learning models in SAR image analysis require large labeled datasets for accurate analysis [98,133]. Researchers have noted that data availability is a significant issue [123]. Obtaining sufficient, high-quality SAR data for training is challenging, and limited data can hinder effective model training. Insufficient data may cause models to miss important SAR imagery features, resulting in poor analysis performance. Addressing this issue is crucial for enhancing SAR image analysis using deep learning. Cao et al. [123] highlighted the urgent need to address the limitation of available PolSAR datasets and suggested using customized data augmentation strategies. Generative models like Generative Adversarial Networks (GANs) can help mitigate the lack of labeled SAR data by generating various SAR data under different conditions (seasons, weather, and sensor parameters). This augmented data can enhance deep learning models’ ability to learn robust features and improve SAR image analysis performance. Although few researchers have used GANs in remote sensing, they have achieved impressive results. Future work should focus on hybrid models combining GANs with deep learning to leverage the strengths of both approaches.

C. Edge Errors and High Computational Time

Inaccurate boundary detection is a major challenge in SAR image analysis, often resulting in edge errors and affecting overall accuracy. Additionally, existing deep learning models for SAR images are often computationally expensive and memory-intensive, making real-time implementation difficult. Graph-based deep learning models, such as GCNs, show significant potential in remote sensing tasks by utilizing spatial and structural dependencies in SAR images. Unlike traditional CNNs that operate on pixel grids, graph-based models work on graph-structured data [123]. A graph-based deep learning model like GCN can work on superpixel-based nodes instead of pixel-based nodes. Superpixels, groups of adjacent pixels with similar characteristics (colour, texture, and intensity), are represented as graph nodes, with relationships modeled as edges. This superpixel-based graph convolutional network (GCN) method reduces edge errors and computational time and enhances the modelling of global contextual relationships in SAR image analysis by leveraging the spatial relationships between superpixels and capturing information around edges. Future work should focus on integrating GCNs as a superpixel-based graph method with CNNs as a pixel-based method, enabling the fusion of local pixel features from CNNs with superpixel features from GCNs. This integration can enhance SAR image analysis by reducing edge errors and lowering computational time.

D. Model Adaptability

Many existing models lack flexibility and adaptability to different types of SAR data and conditions, limiting their applicability across diverse scenarios. Deep learning-based superpixel models in SAR image analysis often cannot adaptively select the superpixel scale, as seen in [125]. Adaptive Graph Convolutional Networks, which allow the graph structure and convolutional operations to adapt based on the data, offer better flexibility in handling SAR imagery’s unique challenges, including varying resolutions, speckle noise, and diverse terrain features. Integrating Adaptive GCNs with deep learning models enhances flexibility and performance by allowing the network to adjust parameters based on the graph’s characteristics. Adaptive GCNs are suitable for various SAR image analysis tasks due to their graph structure adaptation, parameter learning, hierarchical learning, flexibility, and performance improvement. Future work should focus on integrating Adaptive GCNs with deep learning models to improve the accuracy and efficiency of SAR image analysis.

E. Temporal Changes

Temporal changes in SAR images result from differences in images captured at different times, reflecting changes in the environment, objects, or activities. These changes pose significant challenges in SAR image analysis due to the nature of SAR imaging and the complexity of analyzing temporal changes over time. Wang et al. [64] utilized a weighted graph convolutional network to extract multiscale superpixel features, enhancing spatial information utilization in SAR images. However, the model struggles to fully capture the complexity of interactions between nodes when graph edges represent multiple types of relationships. Integrating a multi-weighted graph convolutional network with a deep learning network can enhance SAR image analysis by recognizing features at multiple scales, improving overall performance, and promoting a better understanding of SAR data. This proposed model captures multiscale and multi-dimensional spatial features, particularly improving classification accuracy. Integrating a multi-weighted graph convolutional network with a deep learning network can handle temporal changes and multi-polarization data, improving tasks like change detection, object detection, and anomaly detection in SAR imagery.

For SAR image denoising, this integration enhances image quality by modeling noise characteristics as multi-weighted edges, with each edge weight representing a different noise source. Multi-weighted graph convolutional networks can separate noise from SAR images, facilitating the extraction of meaningful features for analysis. Utilizing these networks with deep learning models will significantly enhance SAR image analysis in applications like disaster monitoring, environmental surveillance, and military intelligence. Future work should focus on this integration.

F. Real-Time Implementation

Advancements in SAR image data processing have increased the demand for real-time SAR image analysis, which is crucial for time-sensitive applications like defense, disaster monitoring, and surveillance. Implementing SAR image analysis models in real-time scenarios is challenging due to the high computational requirements, which arise from factors such as model complexity, large input data sizes, and the need for rapid processing and decision-making. Only a few researchers [134,135,136] have explored real-time implementation of SAR image analysis due to challenges. Developing models for fast, efficient, real-time SAR image processing while maintaining accuracy is necessary.

Future models should optimize real-time performance using lightweight deep learning models like MobileNet, SqueezeNet, and EfficientNet or edge computing models to enhance SAR data processing in resource-constrained environments. These lightweight models balance high performance with low computational requirements, enabling faster, more accurate analysis. Pruning techniques can be explored to improve inference speed on edge devices by reducing model complexity and computational costs. Furthermore, deploying models on FPGA-based hardware, a configurable integrated circuit programmable after manufacturing, could be investigated to support deep learning in resource-constrained environments. Future work should focus on the real-time implementation of SAR image analysis by integrating lightweight deep learning models, applying model pruning techniques, and leveraging FPGA-based hardware into the processing pipeline.

## 6. Conclusions

Synthetic Aperture Radar (SAR) image analysis is a critical area within remote sensing. While deep learning (DL) models have gained significant attention for their ability to automatically learn complex features and demonstrate strong performance, a comprehensive review of their application across the entire SAR processing chain is lacking. This paper provides the first comprehensive overview that encompasses the four key tasks of SAR image analysis: despeckling, segmentation, classification, and detection. Our study reveals that while Convolutional Neural Networks (CNNs) are the predominant choice among researchers, other powerful models like Generative Adversarial Networks (GANs) and Graph Neural Networks (GNNs) remain significantly underutilized. Furthermore, to address the practical challenge of accessing curated datasets for deep learning, this work compiles and provides direct links to commonly used SAR datasets. Finally, we discuss prevailing challenges and outline promising future research directions, offering valuable insights to advance the field of SAR image analysis

## Figures and Tables

**Table 1:** Different SAR bands and their applications [1].

Band	Frequency	Wavelength	Typical Application
X	8–12 GHz	0.038–0.024 m	High-resolution urban monitoring
C	4–8 GHz	0.075–0.038 m	Global mapping, change detection, and monitoring of regions with sparse to moderate vegetation cover.
S	2–4 GHz	0.15–0.075 m	Agricultural monitoring using SAR-based Earth observation
L	1–2 GHz	0.30–0.15 m	Geophysical surveillance and biomass and vegetation assessment
P	0.3–1 GHz	1–0.30 m	Vegetation mapping and biomass monitoring

**Table 2:** Summary of various recent (2020–2024) survey papers in SAR image analysis.

Year	Paper	Title	Focus of Work
2020	[10]	Synthetic aperture radar image classification	Focused on identifying available techniques in SAR image classification.
	[11]	Classification of SAR and PolSAR images using deep learning	Focused on determining deep learning methods suitable for SAR or PolSAR image classification tasks. Standard datasets used in SAR image classification were also presented, and the classification results were presented.
2021	[12]	Complex-valued neural networks for synthetic aperture radar image classification	Focused on complex neural network techniques used in SAR image classification as applied to military targets. Discussed the benefits of each method and presented the accuracy achieved with limited training data, as well as when there is a domain mismatch between the training and testing data.
	[13]	Overview of trends and perspectives in deep learning techniques for despeckling synthetic aperture radar images	Focused on critically analysing the existing methods to identify the most promising deep learning techniques for SAR despeckling. Also, the various factors affecting the success of deep learning techniques were identified.
	[6]	Techniques for SAR image denoising using convolutional neural networks	Focused on image denoising challenges tackled by CNN techniques with various datasets. Compared various SAR image classification techniques and provided potential hybrid models to improve SAR image classification.
	[14]	Deep learning meets SAR	Focused on introducing the most relevant deep learning technique, stated the challenges and achievements. Recommendation of some future research works.
2022	[9]	Applications of convolutional neural networks in synthetic aperture radar, recent advances	Focused on reviewing the major areas of SAR data analysis addressed by convolutional neural networks. Complex-valued convolutional neural networks were also investigated for their capacity to utilize phase information included in SAR complex images.
	[7]	Deep learning for SAR ship detection	Focused on presenting advancements in deep learning algorithms for ship detection in SAR imagery. Highlighted the dataset, algorithm, performance metrics, and deep learning framework used in SAR ship detection. Evaluated the benefits and drawbacks of speed and accuracy.
	[5]	SAR image classification	Focused on discussing, comparing, and highlighting the advantages and disadvantages of different SAR image classification techniques.
2023	[8]	SAR ATR in deep-learning Era	Focused on algorithms for SAR automatic target recognition (ATR). Provided a summary of the frequently utilized datasets and the evaluation metrics. Presented the methods prior to deep learning and SAR automatic target recognition techniques in the deep learning era. Identified the non-CNN and CNN methodologies employed in SAR ATR and outlined prospective directions.
	[2]	Synthetic aperture radar image analysis based on deep learning: A review of a decade of research	Focused on diverse methodologies and architectures for various synthetic aperture radar image applications, presented the target detection and recognition models together with their workflows to assess the methods and performance of these models. Highlighted the merits and demerits of various methodologies to guide other researchers about how different techniques can affect performance for future adoption while also suggesting viable future approaches and hybrid models.
2024	[15]	Deep learning techniques for SAR Image restoration	Focused on the challenges posed by the speckle phenomenon. Highlighted the advancements in speckle reduction methods alongside image restoration methodologies. Discussed the deep learning approaches that have demonstrated superior restoration performance compared to traditional methods.
	[16]	Recent advances in SAR image analysis using deep learning: Examples of speckle denoising and change detection	Focused on recent advancements in the application of deep learning for SAR image analysis, particularly in speckle denoising and change detection.
2025	[17]	Recent advances in deep learning-based SAR image target detection and recognition	Focused on the application of deep learning methods for target detection and recognition in SAR imagery.
	[18]	Review of SAR automatic target recognition: A dual perspective on classical and deep learning techniques	Focused on SAR ATR, spanning: classical and modern approaches.
	[19]	Review of deep learning-based SAR Image ship interpretation Technology	Focused on recent deep-learning-based methods for ship detection and interpretation in SAR imagery, highlights datasets, method types, challenges and future research directions.
	[20]	Fifty Years of SAR Automatic Target Recognition: The Road Forward	Focused on 50-year review of SAR automatic target recognition, analyzing the progression from traditional methods to deep learning, synthesizing recent physics-guided approaches, and compiling publicly available datasets and code resources.

**Table 5:** Commonly used models and their suitability for SAR tasks.

DL Models	Despeckling	Segmentation	Classification	Detection
CNN	Highly ✓	Highly ✓	Highly ✓	Highly ✓
RNN	⊠	⊠	Limited ✓	⊠
DBN	Moderately✓	Moderately✓	Moderately✓	Moderately ✓
AE	✓	Moderately✓	Limited ✓	Limited ✓
GAN	Highly ✓	Moderately✓	Limited ✓	Limited ✓
GNN	Limited ✓	Moderately✓	✓	✓
TRANSFORMER	Limited ✓	Highly ✓	Highly ✓	Moderately✓

Notes: ✓ indicates the DL model is suitable for the SAR task; ⊠ indicates it is not suitable.

**Table 6:** Summary of available datasets for SAR Image Analysis.

Dataset	Band Type	Suitable Tasks	References	Dataset Link
AIRSAR’s flevoland dataset	L	Object Classification and Detection	[52]	https://github.com/fudanxu/CV-CNN/blob/master/README.md (accessed on 2 December 2024)
MSTAR dataset	X	Target Recognition, image classification	[10,12,37,65,118,126]	https://www.sdms.afrl.af.mil/index.php?collection=mstar (accessed 3 October 2024)
OpenSARShip dataset	C	Object detection, scene classification	[47,73,127]	https://opensar.sjtu.edu.cn/DataAndCodes.html (accessed on 1 May 2024)
SARFish Dataset	C	Object detection, scene classification	[53,120]	https://huggingface.co/datasets/ConnorLuckettDSTG/SARFish https://iuu.xview.us/download-links (accessed on 30 May 2024)
FUSAR-Ship dataset	X	Image classification, Detection	[128]	http://www.emwlab.fudan.edu.cn/resources/main.psp (accessed on 3 October 2024)
SARShip Detection Dataset	C	Object Classification, Segmentation and Detection	[122]	https://drive.google.com/file/d/1glNJUGotrbEyk43twwB9556AdngJsynZ/view?usp=sharing (accessed on 3 May 2024)
San Francisco Bay dataset	L	Object detection	[62,129]	https://github.com/anderborba/Code_GRSL_2020_1/blob/master/Data/SanFrancisco_Bay.mat https://github.com/liuxuvip/PolSF (accessed on 4 October 2024)
TerraSAR-X dataset	X	Object detection, scene classification	[33,51]	https://esatellus.service-now.com/csp?id=dar&dataset=TerraSAR-X https://earth.esa.int/eogateway/missions/terrasar-x-and-tandem-x/sample-data (accessed on 3 October 2024)

## Data Availability

Data sharing not applicable.
